# Assessment of school readiness and the importance of executive functions for learning

**DOI:** 10.1590/1984-0462/2024/42/2022196

**Published:** 2023-08-25

**Authors:** Rachel Mocelin Dias Coelho, Sandra Josefina Ferraz Ellero Grisi, Alexandra Valéria Maria Brentani, Ana Paula Scoleze Ferrer

**Affiliations:** a Universidade de São Paulo, São Paulo (SP). Brazil.

**Keywords:** School, Executive function, Literacy, Learning, Child development, Escola, Função executiva, Alfabetização, Aprendizagem, Desenvolvimento infantil

## Abstract

**Objective:**

Considering the importance of the beginning of the academic trajectory for children to reach their full development, this work aims to evaluate the school readiness of preschool-age children and identify which factors influence these results, in order to contribute to the proposition of strategies that allow improving the teaching-learning process and child development.

**Methods:**

This is a cross-sectional, descriptive and analytical study with 443 preschool children belonging to the West Region Cohort (ROC Cohort), from the public school system of the city of São Paulo. School readiness was assessed by the International Development and Early Learning Assessment (IDELA) tool. Non-parametric techniques were used for the correlation analysis between IDELA scores and sociodemographic and socioeconomic conditions: Spearman's parametric correlation, Mann-Whitney and Kruskal-Wallis tests.

**Results:**

The children's mean age was 69 months (standard deviation — SD=2.8; ranging from 55 to 72 months) and most of them came from families with low socioeconomic level. Most children showed adequate readiness in the overall score (65%) and in most domains, except for emergent literacy, in which most (56.9%) were classified as “emergent”. The highest percentage of insufficiency was identified in executive functions (4.1%), which showed a correlation only with the caregiver's education.

**Conclusions:**

Children had adequate school readiness scores, except for emergent literacy, but the insufficiency in executive functions may compromise the future schooling of these children. Thus, pedagogical proposals should consider these aspects for learning and pediatricians need to reinforce the habit of reading and playing games to stimulate child development.

## RESUMO

**Objetivo:**

Considerando-se a importância do início da trajetória acadêmica para as crianças alcançarem o seu pleno potencial de desenvolvimento, este trabalho tem como objetivo avaliar a prontidão escolar de crianças em idade pré-escolar e identificar que fatores influenciam esses resultados, com a finalidade de propor estratégias que possam melhorar o processo de ensino-aprendizagem e o desenvolvimento da criança.

**Métodos:**

Trata-se de um estudo transversal, descritivo e analítico, com 443 pré-escolares pertencentes à Coorte da Região Oeste (Coorte ROC) da rede pública de ensino da cidade de São Paulo. A prontidão escolar foi avaliada pela ferramenta International Development and Early Learning Assessment (IDELA). Técnicas não paramétricas foram utilizadas para a análise de correlação entre escores de IDELA e as condições sociodemográficas e socioeconômicas: correlação paramétrica de Spearman, testes de Mann-Whitney e Kruskal-Wallis.

**Resultados:**

A média de idade das crianças foi de 69 meses (desvio padrão — DP=2,8; variando de 55 a 72 meses) e maioria era proveniente de famílias com baixo nível socioeconômico. A maioria das crianças apresentou prontidão adequada na pontuação geral (65%) e na maior parte dos domínios, com exceção da pré-escrita, na qual as crianças foram predominantemente (56,9%) classificadas como “emergentes”. O maior percentual de insuficiência foi identificado nas funções executivas (4,1%), apresentando correlação apenas com a formação do cuidador.

**Conclusões:**

As crianças apresentaram escores adequados de prontidão escolar, exceto para a pré-escrita, mas a insuficiência nas funções executivas pode comprometer a escolaridade futura dessas crianças. Assim, as propostas pedagógicas devem considerar esses aspectos para a aprendizagem, e os pediatras precisam reforçar o hábito de ler e dos jogos e brincadeiras para estimular o desenvolvimento infantil.

## INTRODUCTION

A child's opportunities and early experiences can strongly affect their learning and academic life. While the first thousand days of life are fundamental to the foundations of brain architecture, the preschool years are the time when neural networks interconnect, allowing the acquisition of important skills such as motor coordination, information processing capacity, understanding and expression of ideas, intentions and emotions and development of executive functions.^1^

School readiness is a complex concept and can be defined as the condition in which a child displays the skills necessary to engage in learning experiences at school and covers several domains, including: physical health and sensorimotor development, communication skills, executive functions, translated into social competence and emotional maturity, general knowledge and cognition, as well as enthusiasm and curiosity to learn.^2^ For school readiness, the child must have neuropsychomotor conditions to learn and must be inserted in an environment that promotes and stimulates learning. Thus, in addition to the child's own characteristics, the family context and the school environment are fundamental for the teaching-learning process and, therefore, several factors can influence and interfere with children's school readiness.^2^

The benefits of early childhood education go beyond child development, extending to the family and society, reducing social exclusion, increasing family income and favoring gender equity, by allowing women to enter the labor market. Studies of adverse childhood experiences have revealed that various factors causing toxic stress (e.g. emotional or physical abuse, chronic neglect, exposure to violence) can result in changes in brain circuitry with subsequent negative effects on physical and mental health. On the other hand, it has been observed that early childhood education programs can minimize the effects of toxic stress on children, in addition to reducing the disparity resulting from socioeconomic inequities.^2^ Early childhood education, as it is the beginning of the school trajectory, has an important role and has as one of the main objectives to prepare the child for entry into elementary education.

The Brazilian Common Core Curriculum (BNCC) developed by the Ministry of Education regulates early childhood education in Brazil. These guidelines were established with the objective of directing the organization of pedagogical proposals, guiding public policies and the planning of early childhood education curricula.^3^

There is a growing concern about which pedagogical strategies are most effective, how they can be implemented considering different contexts and how to assess the quality of early childhood education.^4^ Campos et al. evaluated the quality of education in 147 day care centers and preschools in six Brazilian capitals and found levels of quality considered unsatisfactory, particularly regarding the activities developed, personal care offered and structure of programs offered in preschool.^5^ We do not, however, have more recent data on our children's school readiness.

Considering the importance of the beginning of the academic trajectory for children to reach their full development, this work aimed to evaluate the school readiness of preschool-age children, their performance in each of the domains that make up school readiness and to identify which factors influenced these results, in order to contribute to the proposition of strategies that allow improving the teaching-learning process and child development.

## METHOD

This is a cross-sectional study with children belonging to the West Region Cohort (ROC Cohort). The ROC Cohort was started in 2012 and aims to better understand the relationship between exposure to risk and adversity in early life and long-term outcomes, comprising children living in the Butantã/Jaguaré region, in the west of São Paulo, born at Hospital Universitário de São Paulo (HU-USP), between April 1^st^, 2012, and March 31, 2014. This cohort involves a population characterized by low socioeconomic status and exposure to unfavorable conditions.

During the enrollment period, 7,066 births were registered at the hospital, but 859 of these children were excluded from the cohort because they did not reside in the Butantã/Jaguaré region and 45 were stillbirths, resulting in 6,162 children belonging to the cohort, of which 38 died before three years of age. Between 2018 and 2019, a new wave of follow-up was started, with 1,776 children; however, due to the pandemic in early 2020, field activities were suspended and only 443 children underwent the school readiness assessment, composing the sample of the present study. All the children studied were preschool students from the public school system of the city of São Paulo.

Mothers were invited to participate in the postpartum period, during hospitalization in rooming-in at HU-USP. After inclusion in the cohort, the children were evaluated at six months and, later, at one, three and six years of age, in home visits carried out by contracted and previously trained community agents. The database of this study is composed of the information obtained in the visit carried out at preschool age, which comprises the sociodemographic and socioeconomic data of the families and the assessment of children's development, including the school readiness assessment through the International Development and Early Learning Assessment (IDELA) tool.^6^

IDELA was developed in 2014 by Save the Children, a non-governmental organization that defends the rights of children in the world. The instrument was developed with the aim of supporting the continuous improvement of the program in the various Save the Children and partner countries and has already been used in more than 70 countries, including Latin America. In the validation process in Brazil, IDELA proved to be accessible, applicable in different contexts, feasible and respected for its psychometric rigor, presenting content validity, adequate internal consistency and inter-examiner reliability, and it is an important tool to assess preschoolers’ development.^7^

IDELA is applied at preschool age (between 3.5 and 6.5 years of age) and is based on direct assessment of the child. It is easy to apply, freely accessible and free of charge. It consists of 22 main items that assess gross and fine motor development, emergent numeracy and the ability to apply simple numerical concepts, emergent literacy and socio-emotional development. These domains are scored separately and form an overall score. In addition, the instrument assesses the development of executive functions. IDELA results can be interpreted as continuous (scores) or categorical variables, according to the percentage of correct answers obtained in each domain: insufficient (<25% of hits), emergent (25–74% of hits) and adequate (≥75% of hits).^8^

The independent variables studied were the sociodemographic conditions of the child and main caregiver and the socioeconomic characteristics of the families, namely: child's age, child's gender, gestational age, age at day care, attending preschool, primary caregiver, age of the caregiver, the caregiver's education, the caregiver's marital status and the family's economic classification. The dependent variables were the scores obtained in IDELA in the domains divided into: overall score, motor, emergent literacy, emergent numeracy, socio-emotional and executive functions.

Categorical variables will be described according to their frequencies in absolute and relative numbers (percentages), and continuous variables will be presented as means and standard deviations. Non-parametric techniques were used for the correlation analysis between IDELA scores and independent variables, as the normality of the data was rejected by the Kolmogorov-Smirnov test. Thus, for continuous variables, Spearman's parametric correlation analysis was performed and, for categorical variables, the non-parametric Mann-Whitney and Kruskal-Wallis tests. Analyses were performed using the Stata 12 statistical software package.

Children whose guardians agreed to participate after agreeing and signing the consent form (ICF) were included in the study. The work was presented and approved by the Research Ethics Committee, number of the Certificate of Presentation for Ethical Consideration — CAAE 01604312.1.0000.0065.

## RESULTS

Of the 443 children analyzed, 227 (51.2%) were female and 216 (48.8%) were male. The mean age was 69 months (SD = 2.8; 55 to 72 months). Most children were born at term (93.2%).

The mother was the main caregiver in 78.1% (346) of the cases. The caregiver's mean age was 35 years (standard deviation — SD=9.7; 20 to 71 years) and more than half (56.4%) had completed high school. As for the marital status of the main caregiver, 63.2% were married or lived with a partner. Most families were classified as class C1 or C2 (76%).

**Figure 1:**
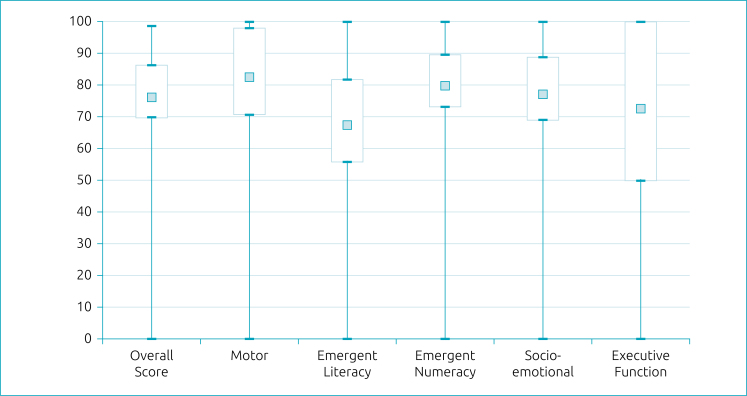
Box-plot of International Development and Early Learning Assessment scores by domain (n=443).

Table 1 and Figure 1 show the IDELA scores and the distribution of means and standard deviation by domain evaluated. It is verified that the pre-written domain presented the lowest scores and the executive functions, the greatest variability in the scores (standard deviation 26.0).

**Table 1:** Mean and standard deviation of International Development and Early Learning Assessment scores by domain.

Domain	Minimum	Maximum	Median	Average	Detour-pattern
Overall score	0	99	79,	76.4	13.4
Motor	0	100	87.7	82.6	18.7
Emergent literacy	0	100	70.0	67.6	19.2
Emergent numeracy	0	100	83.0	79.9	14.0
Social-emotional	0	100	81.0	77.2	16.5
Executive functions	0	100	75.0	72.8	26.0

**Figure 2:**
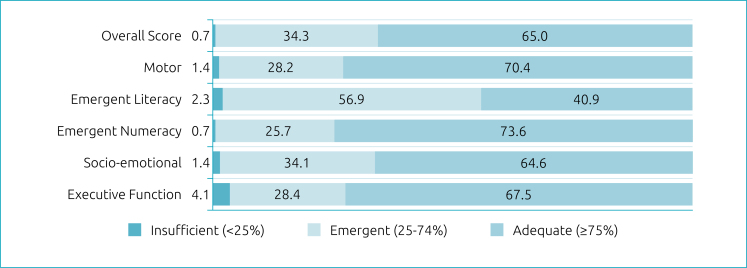
Frequency of readiness classification in each domain (n=443).

Most children showed adequate readiness in the overall score (65%) and in most domains, except for emergent literacy, in which the majority (56.9%) were classified as “emergent”. The highest percentage of insufficiency was identified in the executive functions (4.1%) (Figure 2).

In the analyses it was found that the overall score and the scores in the motor, emergent literacy and emergent numeracy domains were only correlated with the child's age (Table 2). In the evaluation of the socio-emotional domain, no correlation was observed with any of the variables studied. The score for executive functions showed a correlation only with the caregiver's education, with children whose caregivers had “none/elementary school” presenting worse performance than the others (Table 3).

**Table 2:** Association between scores in the domains of school readiness and children's characteristics.

		Average (min-max)	Overall score		Motor		Emergent literacy		Emergent numeracy		Socio-emotional		Executive functions	
			CC	p-value	CC	p-value	CC	p-value	CC	p-value	CC	p-value	CC	p-value
Child's age (months)		69 (55–72)	0.177	<0.001*	0.221	<0.001*	0.128	0.007*	0.158	0.001*	0.077	0.103*	0.037	0.437*
Age at entrance (months)		10,4 (2–12)	-0.018	0.798*	0.075	0.272*	-0.006	0.928*	-0.045	0.513*	0.065	0.345*	-0.120	0.079*
		**N**	**Mean (SD)**	**p-value**	**Mean (SD)**	**p-value**	**Mean (SD)**	**p-value**	**Mean (SD)**	**p-value**	**Mean (SD)**	**p-value**	**Mean (SD)**	**p-value**
Sex	
	Male	216	75.6 (13.5)	0.203†	81.4 (18.9)	0.114†	65.9 (19.7)	0.088†	80.1 (13.8)	0.597†	76.7 (17.2)	0.964†	71.8 (26.2)	0.345†
	Female	227	77.1 (13.3)		83.8 (18.4)		69.1 (18.7)		79.6 (14.3)		77.6 (15.7)		73.8 (25.8)	
Gestational age														
	<37 weeks	28	74.1 (14.2)	0.348†	79.4 (23.0)	0.590†	62.7 (21.5)	0.225†	79.2 (10.9)	0.387†	75.6 (18.5)	0.706†	70.5 (23.6)	0.459†
	≥37 weeks	415	76.5 (13.3)		82.8 (18.4)		67.9 (19.0)		79.9 (14.2)		77.3 (16.3)		72.9 (26.2)	

SD: standard deviation; CC: correlation coefficient; Age at entrance: age at entrance in the school or in the day care.

* Spearman's correlation,

† Mann-Whitney test.

**Table 3:** Association between scores in the domains of school readiness and caregiver's characteristics and socioeconomic conditions.

		Average (min-max)	Overall score		Motor		Emergent literacy		Emergent numeracy		Socio-emotaional		Executive functions	
			CC	p-value	CC	p-value	CC	p-value	CC	p-value	CC	p-value	CC	p-value
Caregiver's age (years)		35 (20–71)	-0.014	0.765*	-0.002	0.962*	-0.024	0.608*	0.011	0.823*	-0.006	0.906*	0.002	0.971*
		**N**	**Mean (SD)**	**p-value**	**Mean (SD)**	**p-value**	**Mean (SD)**	**p-value**	**Mean (SD)**	**p-value**	**Mean (SD)**	**p-value**	**Mean (SD)**	**p-value**
Maternal education														
	Until 9 years	136	75.6 (12.8)	0.303†	84.2 (16.8)	0.082†	66.8 (18.8)	0.594†	78.3 (14.3)	0.069†	77.6 (15.3)	0.977†	67.5 (25.7)‡	0.002†
	Until 12 years	250	76.1 (14.2)		80.7 (20.2)		67.3 (19.4)		79.8 (14.6)		77.0 (16.6)		74.4 (25.3)‡	
	>12 years	56	79.0 (10.5)		87.4 (14.7)		70.3 (18.8)		83.9 (9.6)		76.5 (18.5)		78.1 (27.8)‡	
Marital status														
	With partner	280	76.0 (14.2)	0.721§	82.4 (18.8)	0.678§	67.5 (19.9)	0.921§	79.4(15.3)	0.940§	76.1 (17.7)	0.192§	73.0 (26.9)	0.443§
	No partner	161	76.9 (11.9)		83.0 (18.6)		67.5 (18.0)		80.7 (11.7)		79.1 (14.1)		72.4 (24.5)	
Economic class														
	A/B	50	77.6 (11.2)	0.188†	84.2 (15.9)	0.908†	70.1 (17.3)	0.143†	82.9 (11.0)	0.119†	74.8 (17.9)	0.203†	75.5 (25.0)	0.406†
	C	337	76.7 (13.6)		82.2 (19.2)		67.9 (19.6)		79.9 (14.1)		77.9 (16.4)		73.1 (25.7)	
	D/E	55	73.4 (13.9)		83.6 (17.9)		63.2 (17.9)		76.8 (15.8)		75.2 (15.7)		68.2 (28.6)	

CC: correlation coefficient; SD: standard deviation.

* Spearman's correlation;

† Kruskal-Wallis test;

‡ different letters denote statistically significant differences;

§ Mann-Whitney test,

## DISCUSSION

Our results show that, in general, children have good school readiness, except for emergent literacy, which may have an impact on the literacy process. Another relevant aspect is the great variability observed on executive functions scores, skills considered essential for learning.

The assessment of school readiness is important because healthy integral development during the first years of life will have a positive effect on the acquisition of new knowledge and adaptation to different environments, leading to good academic and professional performance, as well as personal and economic fulfillment, and having impacts on society. Studies show that the earlier the stimuli to promote child development are carried out, the more cost-effective the interventions will be, hence the importance of the first years of life and preschool education.^9^ Thus, when evaluating school readiness and the factors that impact it, it is possible to propose practical and political measures that improve child development, reducing the number of children who enter school with inadequate early learning experiences, avoiding dropout and school difficulties and favoring long-term academic success.

The overall score of the IDELA score showed that the children studied generally performed well, with an average score of 76.4, and 65% of the children achieved scores classified as adequate. In comparison to other studies that used IDELA, the children evaluated in our study showed better performance. A study carried out in the United States of America showed a mean score of 54 and another, in Bolivia, a score of 46.^10,11^ However, the mean age of the children evaluated in these studies was relatively younger compared to our sample (48, 56 and 69 months, respectively). In addition, socioeconomic and cultural differences between countries can also justify the variations in the scores found.^11^ Possibly, in our study, we had a good overall score due to the mean age of the children studied (69 months), and due to a possible bias effect, since the studied sample received interventions aimed at stimulating child development throughout the cohort.

There was a positive correlation between the child's age and the overall score of school readiness and motor performance in emergent literacy and emergent numeracy, as expected, since the IDELA evaluates children aged 42 to 78 months old in the same way. No association was found with other variables, unlike what was described in other studies, which report an association between school readiness and parents’ age and education and socioeconomic conditions. Positive correlations have been described between child learning and socioeconomic level and parental education, related to the presence of greater support and appropriate stimuli for the child's development.^12–15^ In our study, we did not find this correlation, possibly because we analyzed a more homogeneous population; the sample studied included only children from the cohort, living in the western region of São Paulo and attending public schools, therefore with similar socioeconomic and family educational levels.

In the analysis of scores in the domains that make up the overall score (motor, emergent literacy, pre-mathematical and socio-emotional) we found that the children performed well, except for emergent literacy, whose average score was 67.6, and more than half of the children (56.9%) classified themselves as “emerging”. In Brazil, BNCC defines that literacy must occur until the second year of elementary school, in order to guarantee the fundamental right to learn to read and write. However, this learning requires both cognitive and motor skills (reading and writing), which can be influenced by several factors in early childhood, demonstrating the complexity of the literacy process.^3,16^ A study carried out with American children, also using IDELA, showed unsatisfactory results from children in terms of letter recognition, similar to our results in this domain.^10^

Learning to read and write is a complex process, through which the child learns to decode and encode language into symbols, therefore, it encompasses several aspects: knowledge of the alphabet, acquired vocabulary, phonological awareness, which involves the perception of different sounds that make up the words and notions of impression, that is, the difference between pictures and words and the reading sequence from left to right.^16^ Research has already shown that the habit of reading and the child's verbal interaction with their parents or caregiver have an impact on the development of language and vocalization, helping the child in school readiness and future cognitive development. Reading even for the baby already demonstrates several benefits in the process of language acquisition and linguistic capacity, in addition to promoting affective bonds between parents and children, strengthening the child in the psychic and emotional areas and facilitating that habit for the child.^17^ We do not have information on reading habits in this sample, but possibly the prevalence of reading parents for children is low, if we consider the habits in our environment. According to the publication “Portraits of Reading in Brazil”, a national study, with a sample of socioeconomic level and education similar to ours, the reading habit among Brazilians is very low and, even more worrying, 60% of those surveyed reported that their parents had never read to them.^18^

In recent years, the debate about school readiness and learning has shifted from pre-academic skills and focused primarily on executive functions. Executive functions are fundamental for autonomy, execution of tasks, personal relationships and, therefore, for learning. These are skills used in problem solving, activities aimed at goals, self-control and the ability to retain and manipulate various pieces of information at the same time.^19^ They involve skills related to both taking actions and controlling emotions and responding to situations, and they are didactically divided into: working memory, inhibitory control and cognitive flexibility.^19^ Several authors have proposed that skills related to executive functions are the most important for the academic performance of children and adolescents and, in the future, for professional success. According to Nayfeld et al.,^20^ executive functions significantly predicted gains in math, vocabulary, listening comprehension and science readiness, showing their importance for readiness. Blair and Razza^21^ explained that executive functions are more strongly associated with readiness than intelligence quotient (IQ) and emergent literacy and emergent numeracy skills. Therefore, executive functions have been one of the main focuses of attention in the literature on school performance and readiness and one of the most current concerns in the proposition of curricula and pedagogical strategies.

In IDELA, the executive functions evaluated are the ability to self-regulate and short-term memory, which is considered a separate domain and does not make up the overall score. In our study, the children had a lower mean score than the overall score for the other pre-academic skills (72.8 and 76.4, respectively) and, although 67.5% of them had scores classified as adequate, it was the domain in which the highest percentage (4.1%) was classified as insufficient. Furthermore, the score for executive functions showed the greatest variability in the sample (SD=26.0). This finding is one of the main results of our study: although the analyzed children had adequate school readiness scores, except for emergent literacy, the insufficiency in relation to executive functions may compromise the future schooling of these children. Possibly, if executive functions were included in the overall score in IDELA, our school readiness results would possibly be worse than those observed.

The brain circuits responsible for executive functions show an enormous development between three and five years of age, and although these circuits are refined during adolescence to adulthood, the fundamental connections are established in preschool age.^9^ Thus, a correlation between this score and the child's age was to be expected, but we did not find this result. We found a significant association between the score in executive functions and maternal schooling — children of mothers with less time of schooling performed worse compared to those of mothers with higher education. This finding can be explained by less exposure to situations that stimulate the development of these skills. Experiences such as involvement in games, plays, sports, music, reading are important stimuli for the formation of neural circuits related to executive functions.^22^

Therefore, our findings reinforce the current discussion about different perspectives in relation to school readiness, particularly the importance of early childhood education, including stimuli for the development of affectivity, sociability and executive functions, and not just cognitive skills.^23^ Curricula should consider the importance of playing at this stage of life, which allows sensory exploration, the exercise of impulsive control, focus, attention and memory training, in addition to providing learning in contexts of socio-affective relationships. Thus, through games and play, important characteristics are developed such as cooperation, negotiation and self-control, creativity and imagination,^9^ the most important aspects for learning and for school readiness.

As limitations of our study, we highlight the bias of the population having received interventions to stimulate development during the cohort, in addition to ours being a more homogeneous sample from the socioeconomic and cultural aspects. On the other hand, we emphasize as positive points the number of children evaluated and the sample coming from a lower socioeconomic level and, therefore, possibly more vulnerable, and who can benefit from interventions and public politics aimed at optimizing children's learning, particularly those aimed at the development of executive functions.

Finally, we observed that although the analyzed children presented adequate school readiness scores, the results in relation to emergent literacy skills and executive functions indicate the need for pedagogical proposals to consider these aspects for learning, in addition to the importance of pediatricians reinforcing the habit of reading and playing games to stimulate child development.

## Data Availability

The database that originated the article is available with the corresponding author.
